# Enhancing Infrared Optical Flow Network Computation through RGB-IR Cross-Modal Image Generation

**DOI:** 10.3390/s24051615

**Published:** 2024-03-01

**Authors:** Feng Huang, Wei Huang, Xianyu Wu

**Affiliations:** School of Mechanical Engineering and Automation, Fuzhou University, Fuzhou 350108, China

**Keywords:** optical flow, infrared image, deep neural network

## Abstract

Due to the complexity of real optical flow capture, the existing research still has not performed real optical flow capture of infrared (IR) images with the production of an optical flow based on IR images, which makes the research and application of deep learning-based optical flow computation limited to the field of RGB images only. Therefore, in this paper, we propose a method to produce an optical flow dataset of IR images. We utilize the RGB-IR cross-modal image transformation network to rationally transform existing RGB image optical flow datasets. The RGB-IR cross-modal image transformation is based on the improved Pix2Pix implementation, and in the experiments, the network is validated and evaluated using the RGB-IR aligned bimodal dataset M^3^FD. Then, RGB-IR cross-modal transformation is performed on the existing RGB optical flow dataset KITTI, and the optical flow computation network is trained using the IR images generated by the transformation. Finally, the computational results of the optical flow computation network before and after training are analyzed based on the RGB-IR aligned bimodal data.

## 1. Introduction

The optical flow algorithm leverages pixel changes across a sequence of images in the time domain to analyze and understand the motion of objects moving within the observed imaging plane. This technique holds significant research implications for various fields, including visual navigation and motion inference [1]. The theoretical foundation of the optical flow algorithm relies on the assumption of luminance invariance. This assumption assumes that the luminance values of all pixels in two consecutive images involved in the optical flow computation remain constant over a short period of time. However, this assumption imposes limitations on the general applicability of the optical flow method, as it assumes a specific type of scene and may not hold true in all situations. In recent years, some researchers have also paid more attention to the use of optical flow algorithms in complex scenes, such as low-light dark environments. The essence is mostly to perform richer preprocessing on RGB images to obtain more semantic information [2]. The influence of environmental factors on the imaging of ordinary RGB cameras has also prevented the practical application of most optical research, but the emergence of infrared (IR) technology has been the key to breaking the deadlock to some extent. IR imaging technology itself is not affected by external light, and the use of IR thermal imaging cameras can directly capture the radiation signal emitted from objects to generate human-eye recognizable images with a strong penetrating power [3]. Consequently, exploring the computation of optical flow specifically for IR images can significantly expand the practical applications of optical flow algorithms.

Image optical flow computations are mainly categorized into traditional linear (Lucas–Kanade [4], Horn–Schunck [5], etc.) and deep learning-based (PWC-Net [6], VCN [7], RAFT [8], etc.) algorithms. Among them, deep learning-based optical flow computation algorithms have been shown to be able to obtain a more accurate computational accuracy for RGB images. Deep learning algorithms can extract richer image features by using a stacked convolutional computation module [9,10], which is beneficial for optical flow solving. In contrast, current experiments [11,12,13] involving optical flow computations of IR images still mainly use traditional algorithms or cannot independently use deep learning optical flow algorithms and validation [14]. The reason for this is that the fitting of network parameters for deep learning algorithms requires an optical flow dataset with the true value of the optical flow, which is often costly to produce. Most of the optical flow datasets (FlyingChairs [15], etc.) are generated using synthetic techniques such as PS (Photoshop) and are not derived from real scene acquisition. Only one dataset, KITTI [16], was created by combining RGB cameras with multi-sensors to capture real scene information. In the RGB image domain, existing optical flow datasets are extremely limited, and creating an optical flow dataset in the IR image domain is undoubtedly an even more voluminous project. This directly hampers the application of deep learning algorithms based on the computation of optical flow in IR images.

The first consideration for training optical flow-computing networks that lack scene-specific datasets is often an unsupervised training approach [17]. This approach does not require a dataset with real optical flow data, and it has better flexibility in dealing with more realistic and complex scenarios. Although it is more promising, the overall accuracy is still inferior to supervised networks in optical flow computations [18].

**Figure 1:**
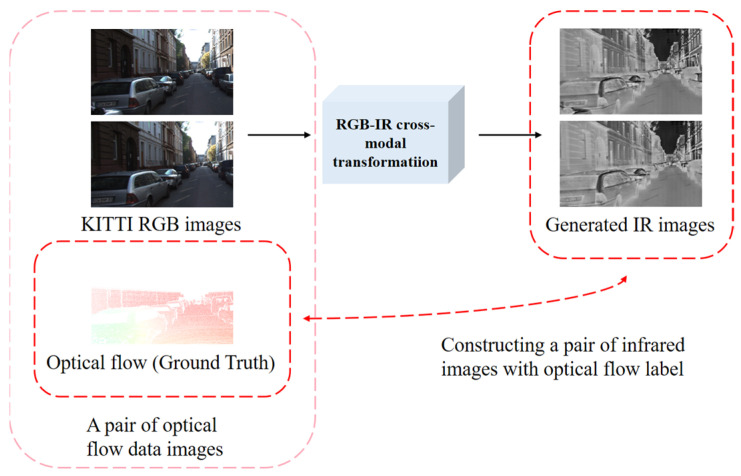
Image (from KITTI) transformed from RGB domain to IR domain.

**Figure 2:**
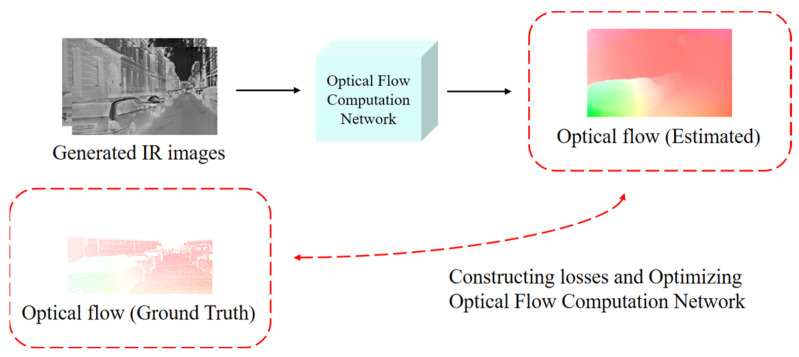
Generated IR images to train the optical flow computation network.

Considering the previously mentioned challenges and practical constraints, we propose a new approach to solve the parameter fitting problem of optical flow computational networks in real IR scenes. As shown in Figure 1, we utilize a deep learning-based image transformation algorithm. We generate IR images from existing RGB optical flow datasets across modalities. Each pair of images in an existing optical flow dataset has two RGB images and one optical flow map. As shown in Figure 2, we utilize the IR images obtained by converting two RGB images using the optical flow map to train the optical flow computation network. This can improve the optical flow computation accuracy of the optical flow computation network in IR scenes.

The main contributions of this work can be summarized as follows:

(1) Achieving cross-modal image transformation between RGB and IR. Drawing upon research on style transfer and image-to-image networks, this paper proposes a redesigned RGB-IR cross-modal image transformation network.
(2) Realization of optical flow computational networks for fine-tuning in IR scenes. In this paper, all the RGB images in the KITTI optical flow dataset are converted to IR images. Subsequently, the converted IR images are employed to train the optical flow computation network, thereby enhancing its performance, specifically for IR scenes.

## 2. Related Work

Currently, there are still relatively few optical flow studies based on the IR domain, especially in the field of deep learning algorithms. One of the most important reasons is the lack of optical flow datasets and the difficulty in producing them. However, the lack of IR datasets is not only a problem in the field of optical flow, but also in the field of IR identification, where only some research tools are worth learning from. As mentioned in AlignGAN [19], RGB-IR cross-modal image transformation operations can be performed based on GAN. Simply speaking, this method utilizes algorithms to convert real RGB images into fake IR images and utilizes high-quality IR image generation to extend the dataset.

Therefore, our rationale is the same. However, the slight difference is that the experiments in AlignGAN are centered around RGB-IR cross-modal pedestrian identification, and the experimental dataset is focused on the SYSU-MM01 dataset [20]. In contrast, our experiments are focused more on optical flow computation. There is one currently available real RGB optical flow dataset, KITTI, a dataset dominated by real street scenes. In the optical flow computation experiments, KITTI is the labeled dataset. But in the RGB-IR cross-modal transformation experiments, KITTI is the unlabeled dataset. There are often two kinds of operations performed on unlabeled datasets. One is to use an unsupervised network such as CycleGAN [21] to train and process the images. However, this method tends to be too random, and the generated images are most of the time unsatisfactory or even excessively distorted. The other is to use similar or similarly labeled datasets to supervise the training of the network and then use the strong generalization ability of the deep learning algorithms to achieve transformation of the unlabeled datasets. This method is easier to train and produces better images because of the exact convergence object, but the performance on unlabeled datasets is more of a test of the network’s own generalization ability.

After significant observation, we found that the M^3^FD [22] dataset provided by the Dalian University of Technology has many similarities with the KITTI dataset in terms of scenes and is based on a larger range of live street scenes. M^3^FD is an aligned RGB-IR bimodal dataset. Therefore, we propose to use M^3^FD for supervised training of image transformation networks to obtain more accurate and higher quality modal transformations.

Supervised image transformation can be traced back to the classical model of Pix2Pix [23]. The article states that the U-Net [24] structure of the model is more favorable for image generation. In addition, the use of pixel consistency loss is able to approximate the generation of low-frequency information in the image, while the high-frequency information is optimized using the generative adversarial loss (Patch Gan as the discriminator).

## 3. Proposed Method

In this paper, the research includes both RGB-IR cross-modal image transformation and optical flow computation networks that are fine-tuned for IR scenarios.

**Figure 3:**
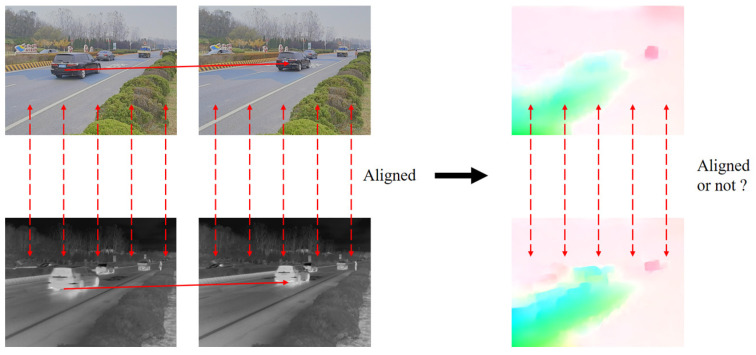
Aligned RGB-IR image optical flow is also aligned.

The RGB and IR images are shown in Figure 3, with the RGB and IR images aligned at moments t and t+1. It is well known that the definition of optical flow is a description of the motion of a flat imaging object. The aligned RGB and IR images have the same image object source without considering special cases such as masking. As shown in Figure 3, the displacement of the car in the IR image is consistent with that in the RGB image. The motion of the flat imaging object is consistent to the above theory and can be regarded as aligned RGB and IR images corresponding to the real optical flow, which is also consistent.

Therefore, it is feasible for us to convert RGB images from existing optical flow datasets into IR images and then train the optical flow computation network using the generated IR images and real optical flow maps of RGB.

### 3.1. RGB-IR Cross-Modal Transformation

**Figure 4:**
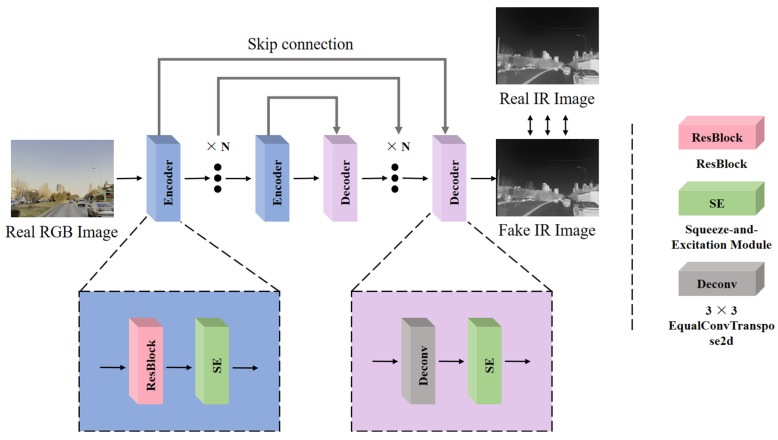
RGB-IR cross-modal transformation of an image.

As shown in Figure 4, the image transformation network in this paper adopts a Pix2Pix-style supervised training architecture, where we use the labeled dataset M^3^FD for supervised training of the network. Supervised training enables the network to learn the relationship between the RGB and IR domains of the input images. This learning process helps the network to better understand the data and realize the transformation of RGB to IR images. The network first performs deep feature encoding of the RGB image using residual blocks with the SE (squeeze-and-excitation) [25] module and then recovers and reconstructs the image using transposed convolution. There is some cross information between the RGB and IR domain, and some information barriers exist at the same time. The use of the SE module can help the network to pay more attention to the cross information with the IR domain when feature encoding the RGB image. At the same time, when decoding, the use of the SE module can make the image generation more adaptive.

In the design of the loss function, this paper uses pixel consistency, gradient consistency, and generative adversarial loss to jointly constrain. The pixel consistency loss $L_{pixel}$ uses the L1 loss, which is insensitive to outliers and can promote the network to generate smoother generation results.

(1) $$L_{pixel}=\left\|I_{real}-I_{fake}\right\|$$

The $L_{pixel}$ facilitates the generation of most of the low-frequency information of the image but is weak in the high-frequency information part. Therefore, gradient consistency loss $L_{grad}$ and generative adversarial loss $L_{gan}$ are further used to enhance the recovery of high-frequency information of the image. The gradient consistency loss uses the Sobel operator to compute the gradient of the generated IR image and the real IR image separately and then compares the difference between the two.

(2) $$L_{grad}=\left\|Sobel\;\left(I_{real}\right)-Sobel\;\left(I_{fake}\right)\;\right\|$$

**Figure 5:**
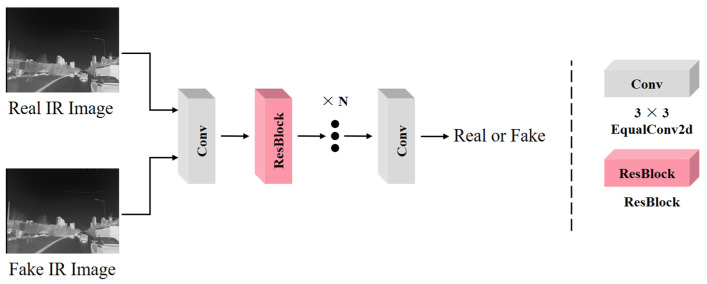
Patch Gan architecture.

For $L_{gan}$, we used Patch GAN to characterize the generated IR image or the real IR image, constraining the network to generate an IR image that is closer to the real one. The image transformation network and Patch GAN are performed in stages. In the Patch GAN training phase, the image transformation network parameters are not updated. Patch GAN inputs the generated IR image and the real IR image, and the constrained Patch GAN determines that the generated IR image is “False” and that the real IR image is “True”. The Patch GAN parameters are not updated during the image transformation network training phase. Patch GAN inputs the generated IR image and determines it to be “True” to adjust the parameters of the image transformation network. The “True” or “False” status of an image is abstracted into a mathematical model, corresponding to “1” or “0”. The more real the input image is, the closer the Patch GAN result is to 1, and vice versa. The structure of Patch GAN is shown in Figure 5, where an H×W image is compressed into a 32×32 feature map using multi-level convolution with residual blocks. Each point on the feature map corresponds to a mapping of a certain region of the original image.

(3) $$L_{discriminator}=\left\|\;1-Discriminator\left(I_{real}\right)\right\|+\left\|Discriminator\left(I_{fake}\right)\right\|$$

(4) $$L_{gan}=\left\|\;1-Discriminator\left(I_{fake}\right)\right\|$$

**Figure 6:**
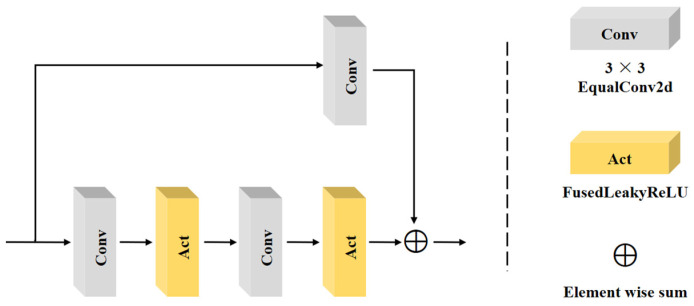
Residual block architecture.

On both the image transformation network and Patch GAN, we use the residual block, whose specific structure is shown in Figure 6. We used EqualConv2d and FusedLeakyReLU to construct the residual block. EqualConv2d keeps the learning rate relatively consistent between different layers of the network, which helps it to better maintain gradient stability during parameter tuning and mitigate problems such as gradient explosion or gradient vanishing. FusedLeakyReLU combines the activation function and the normalization step to reduce the memory footprint and improve the training efficiency of the network.

**Figure 7:**
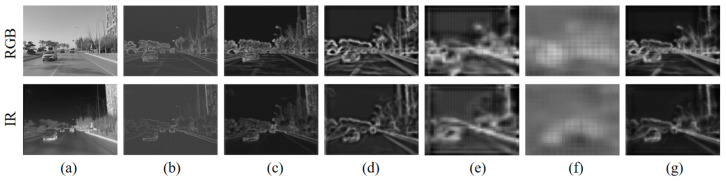
Comparison of RGB-IR bimodal image results for edge detection. (**a**) RGB and IR images to be detected; (**b**–**f**) detection results at different scales, respectively; (**g**) detection results of fusing different scales.

In addition, on the task of transforming unlabeled RGB images, we fine-tune the parameters of the network using the edge consistency loss $L_{edge}$ and perceptual consistency loss $L_{percept}$. First, we use a labeled RGB-IR aligned bimodal dataset to obtain an RGB-IR image transformation network via supervised training. Theoretically, the heavily trained network should generalize on unlabeled RGB images to generate more realistic IR images. In practice, the neural network suffers from a certain degree of underfitting to “things” that have never been seen before. Although the network can map most of the pixels in the image from the RGB to IR domain, the remaining pixels that “fail to map” tend to generate random noise in the generated image, which makes the image distorted. We observe aligned RGB-IR images in an attempt to finetune the network by capturing information about the intersection of the RGB and IR domains. In Chipgan [26], the authors used edge consistency loss to maintain structural similarity before and after image transformation. This method inspired our work. Figure 7 shows the multi-level edge features of the aligned RGB and IR images, respectively. After an extensive analysis, we found that there exists some structural features between the edge images of the RGB and IR images that are more similar than the original images. Therefore, we extracted the multilevel edges of the images using the pre-trained network HED and then constructed the edge similarity loss $L_{edge}$.

(5) $$L_{edge}=\left\|HED\left(I_{rgb}\right)-HED\left(I_{ir\left(fake\right)}\right)\right\|$$

**Figure 8:**
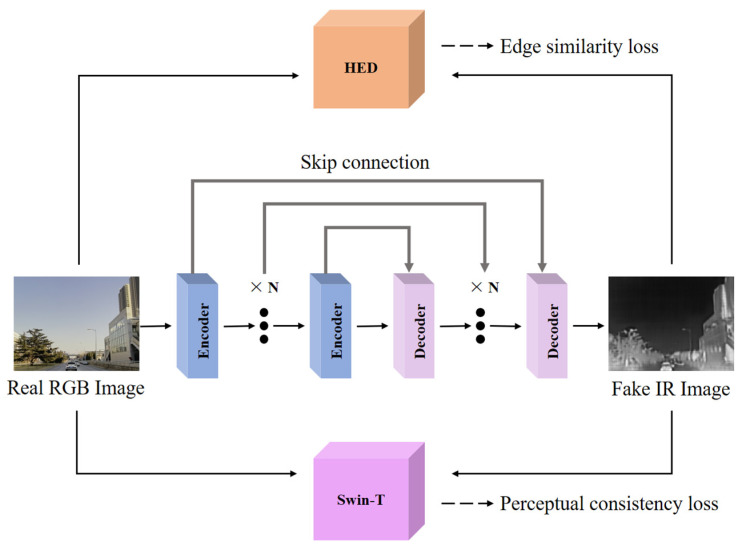
Fine-tuning of unlabeled images.

**Figure 9:**
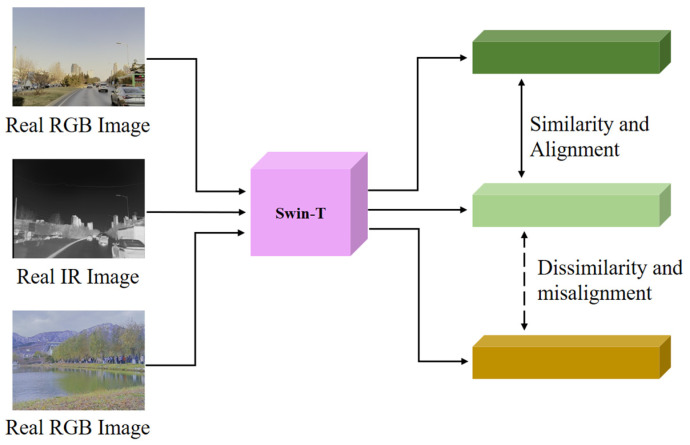
Feature alignment fine-tuning using Swin-T.

As shown in Figure 8, since the edge features of the RGB and IR images are again not exactly aligned, we use the pre-trained Swin-T [27] model to construct a perceptual consistency loss $L_{percept}$ for the image generation. We use the aligned RGB-IR dataset to fine-tune the pre-trained Swin-T, as shown in Figure 9, and we use the triple loss $L_{percept}$ to constrain the Swin-T to extract the aligned feature vectors as much as possible.

(6) $$L_{percept}=relu\left(\left\|Swin\left(I_{rgb1}\right)-Swin\left(I_{ir}\right)\right\|-\left\|Swin\left(I_{rgb2}\right)-Swin\left(I_{ir}\right)\right\|+margine\right)$$

### 3.2. Optical Flow Computation

For the RGB image optical flow computation, RAFT (recurrent all-pairs field transforms) has been proven to be the most accurate algorithm, while FastFlowNet [28] is the leading lightweight optical flow computation network. The parameter size of FastFlowNet is only 1.37 M, and its low parameter count and computational complexity make it possible to further apply the optical flow network in mobile devices. Therefore, this paper discusses the computation of IR images using both RAFT and FastFlowNet.

#### 3.2.1. RAFT

**Figure 10:**
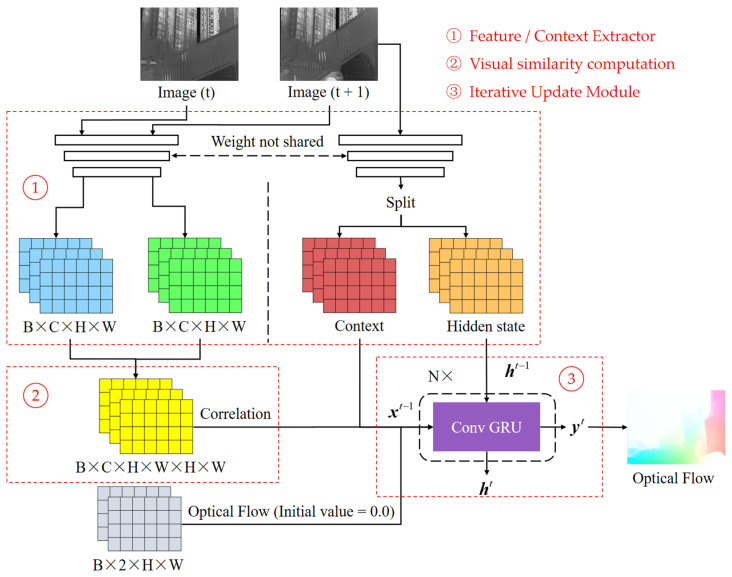
Pipeline of RAFT [8].

As shown in Figure 10, RAFT consists of three main modules: contextual feature extraction, visual similarity computation, and an iterative update module.

Among them, the contextual feature extraction module and the visual similarity computation module are jointly involved in the computation of the visual similarity matrix. The shared weights contextual feature extraction module simultaneously extracts feature information from two frames, forming a pair of feature maps, with each channel corresponding to a specific feature. The inner product of these feature map pairs quantifies the visual similarity between the two frames, with a value closer to 1 indicating a greater similarity of the features. Suppose the size of the feature map involved in the visual similarity computation is H×W, then the size of the inner product calculated is H×W×H×W (four-dimensional tensor). Because the multi-scale image similarity features are more sensitive to the abrupt change capture of motion, RAFT pools the last two dimensions of this tensor using pooling kernels of size 1, 2, 4, and 8, respectively, to construct a multi-scale visual similarity pyramid. After that, using the latest optical flow results obtained from the iterative update module (with an initial value of 0), the domain feature capture radius r is positioned in the corresponding position of this pyramid to construct a visual similarity matrix of size $H\times W\times \left(2\times r+1\right)\times \left(2\times r+1\right)$.

The iterative update module in RAFT employs a convolutional gated recurrent unit (Conv GRU), which is distinct from conventional GRU in that all fully connected modules are replaced by convolutional modules. Additionally, the parameters in the module are shared across each iteration of the Conv GRU, which significantly reduces the number of parameters required. This design enables RAFT to efficiently compute the optical flow results from coarse to fine iterations.

The gating logic of the Conv GRU is divided into four parts:

(7) $$z=sigmod(Conv(\begin{array}{c} x^{t}\\ h^{t-1} \end{array}))$$

(8) $$r=sigmod(Conv(\begin{array}{c} x^{t}\\ h^{t-1} \end{array}))$$

(9) $$h^{\prime }=tanh(Conv(\begin{array}{c} x^{t}\\ h^{t-1}⊙r \end{array}))$$

(10) $$h^{t}=z⊙h^{t-1}+(1-z)⊙h^{\prime }$$

Equation (5) gates z to control the update. $x^{t}$ is the current input containing the latest optical flow iteration results, visual similarity matrix, and contextual feature information (obtained using a separate contextual feature extraction module). $h^{t-1}$ is the state volume passed down from the previous iteration. The activation function sigmoid transforms the data to a value in the range of (0, 1), which determines the retention weight of the state volume. Similarly, gates_*r*_ are used to control the reset specific gravity (Equation (6)). In Equation (7), after the reset $h^{t-1}$ is spliced with $x^{t}$ for one convolution, the activation function tanh is used to deflate the data in the range of (−1, 1) to obtain $h^{\prime }$. In Equation (8), according to the assignment of z, the current stage $h^{t}$ selectively remembers $h^{\prime }$, and the selected forgotten part updates the memory $h^{\prime }$.

#### 3.2.2. FastFlowNet

**Figure 11:**
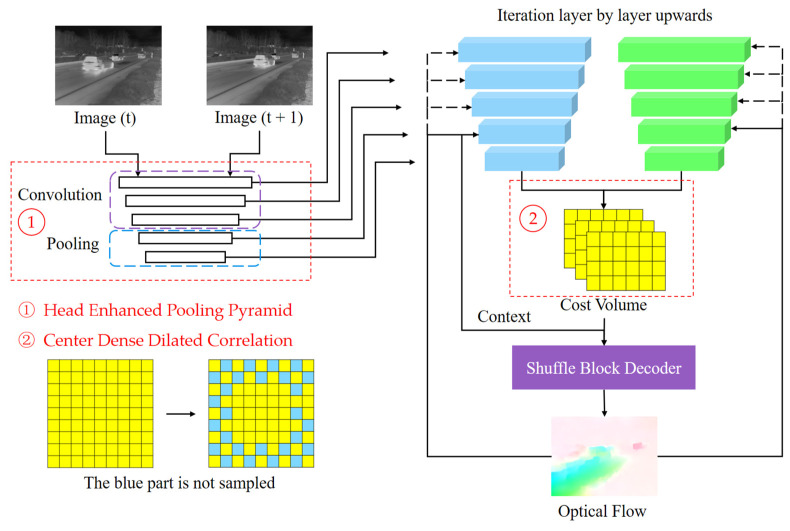
Pipeline of FastFlowNet [28].

As shown in Figure 11, FastFlowNet follows the classical optical flow network PWC-Net pyramid multi-scale computational architecture, with extensive model optimization and parameter pruning in feature extraction, feature correlation computations, and optical flow decoding computations.

In the feature extraction process, head enhanced pooling pyramid (HEPP) is introduced. It retains the convolutional extraction approach of PWC-Net for high-level feature extraction. However, for low-level feature extraction, parameterless pooling extraction is employed instead of convolutional extraction. This design choice reduces the number of convolutional parameters and computational complexity by two layers while still maintaining a reasonable computational accuracy. The optical flow5 is computed from the smallest layer5, and the features of layer4 are mapped according to the computed flow5 to align the two frames. Then, the remaining optical flow of that layer (flow4) is computed for layer4, and so on, iteratively.

The optical flow computation of each layer is obtained by decoding the optical flow from each point of the previous image of that layer. This is achieved by performing feature vector dot product computations on the points of the next image within a specific search radius to construct the image feature correlation cost volume, then decoding the cost volume by overlaying the convolutional or pooling layers. A large number of experiments have shown that expanding the search radius can improve the accuracy of subsequent optical flow computations. However, blindly increasing the radius leads to a significant increase in the computational complexity. To address this issue, FastFlowNet introduces a densely expanded search and acquisition approach with a center dense dilated correlation. This approach resembles dilated convolution, where only the two outermost layers of the search domain undergo feature correlation using cross-interval sampling. The remaining layers are kept fully sampled. This approach constructs a much smaller custom and computational volume than that of a search radius of 4 while achieving a larger feature vector correlation perception without adding excessive parameters or computational complexity.

The pyramid extraction method of HEPP is a layer-by-layer down-sampling extraction method. In contrast, the optical flow needs to be continuously up-sampled in the iterative optical flow back computation from the bottom to the top layer. The design of the up-sampler directly impacts the accuracy of the final optical flow [29]. While complex up-samplers can yield highly accurate up-sampling results, they often overlook considerations related to model parameters and computational complexity. To address this challenge, FastFlowNet introduces a shuffle block decoder based on the ShuffleNet [30,31] lightweight convolutional network. This design ensures high-accuracy optical flow computation while significantly optimizing computational costs. By leveraging the efficiency of ShuffleNet, FastFlowNet strikes a balance between accuracy and computational complexity in the up-sampling process.

## 4. Experimental Results

### 4.1. Experimental Results of RGB-IR Cross-Modal Transition

In this paper, the RGB-IR cross-modal transformation experiments are realized based on the RGB-IR bimodal dataset M^3^FD. First, M^3^FD is divided into a training set and test set in a ratio of 2:1. In our experiments, the generation results of our RGB-IR cross-modal image transformation network are compared with those of real IR images, Pix2Pix, FDIT [32], and AlignGAN2 [33] (unnamed in the original article, recent results from the original AlignGAN team).

**Figure 12:**
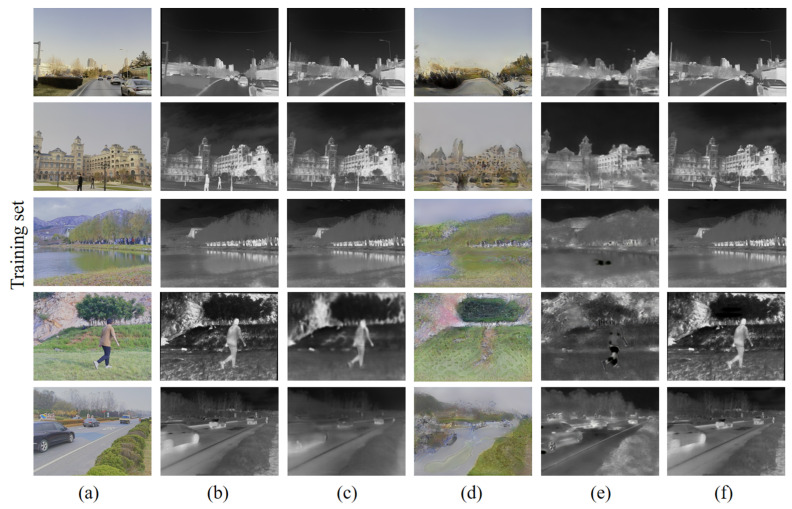
Comparison of RGB-converted IR image results of different networks on the training set: (**a**) RGB images; (**b**) real IR images; (**c**) Pix2Pix; (**d**) FDIT; (**e**) AlignGAN2; (**f**) ours.

The results of the RGB-IR cross-modal image transformation experiments are shown in Figure 12 for each algorithm used on the training set. Visually, the algorithms based on supervised training (ours and Pix2Pix) achieve better convergence results on the training set. The unsupervised training-based FDIT and AlignGAN2, on the other hand, clearly do not converge to better results in the same training period, especially FDIT. FDIT is based on the idea of separating the “content” and “style” of an image and then interacting with the “style”. This idea is theoretically feasible, but in the actual test, it seems difficult to realize the perfect segmentation between the “content” and “style” of the image, resulting in the actual residual “style” in the “content”. However, in practical tests, it seems difficult to realize perfect segmentation between the “content” and “style” of an image, resulting in the residual of “style” in “content”. AlignGAN2, which is essentially an unsupervised algorithm based on CycleGAN, generates images with strong randomness and is conservative in image transformation. Its generation results still have more elements of the original image under close examination.

**Figure 13:**
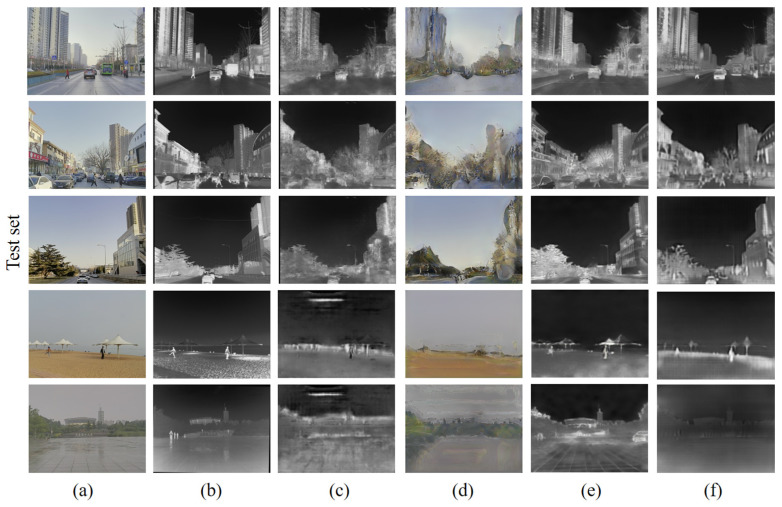
Comparison of RGB-converted IR image results of different networks on the test set: (**a**) RGB images; (**b**) real IR images; (**c**) Pix2Pix; (**d**) FDIT; (**e**) AlignGAN2; (**f**) ours.

The generated results of each algorithm (completed training) on the test set are shown in Figure 13. Visually, the results of each algorithm on the test set have slipped compared to the training results. The two supervised networks with better training results still have more room for improvement when facing the generalization problem. In particular, Pix2Pix, despite perceptually being able to find most of the pixels directly transformed, appears rich in noise, making the image look less smooth and contoured. In contrast, ours performs perceptual and edge fine-tuning on the unlabeled dataset, and the generated results are visually optimized and closer to the real IR images.

**Figure 14:**
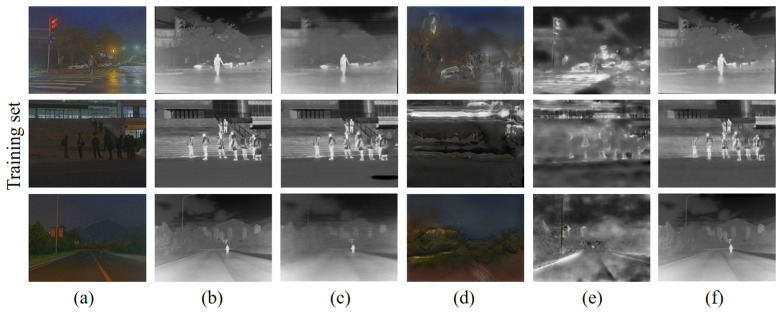
Comparison of RGB-converted IR image results of different networks on the training set: (**a**) RGB images; (**b**) real IR images; (**c**) Pix2Pix; (**d**) FDIT; (**e**) AlignGAN2; (**f**) ours.

Since the unlabeled dataset KITTI is a daytime scene dataset, we mainly used the daytime scene part of M^3^FD when training the RGB-IR cross-modal image transformation network, trying to push the labeled training dataset to be highly similar to the unlabeled dataset to be generalized. Nevertheless, we still focus on the black night scene part of M^3^FD. The training results for the dark night scene are shown in Figure 14, and the supervised network can still form a good convergence for image generation in the dark night scene.

**Figure 15:**
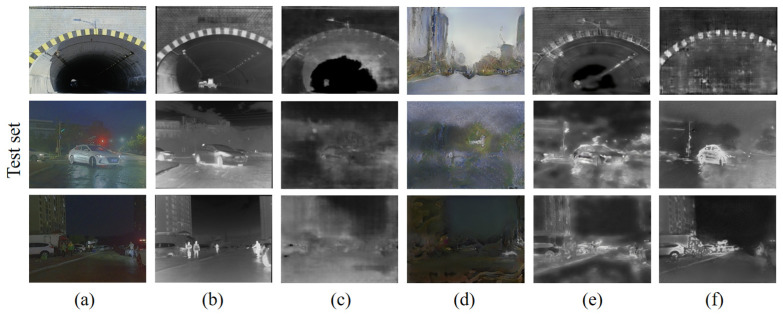
Comparison of RGB-converted IR image results of different networks on the test set: (**a**) RGB images; (**b**) real IR images; (**c**) Pix2Pix; (**d**) FDIT; (**e**) AlignGAN2; (**f**) ours.

However, the results on the test set are not satisfactory. As shown in Figure 15, the visual difference between the RGB and IR images for the nighttime scenes is greater than for the daytime scenes due to bright light or shadow occlusion. IR images tend to have a lot of “things” that are not present in RGB, which may require more algorithmic combinations, such as “things” prediction followed by pixel transformation, when faced with more prediction problems.

**Figure 16:**
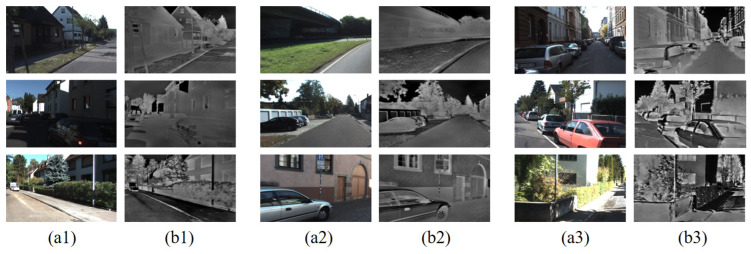
RGB-IR cross-modal transformation (KITTI, unlabeled): (**a1**–**a3**) RGB images; (**b1**–**b3**) generated IR images.

However, the RGB-IR cross-modal transformation capability demonstrated by the algorithm in daytime scenes is sufficient for us to process the KITTI dataset. The generation results of the algorithm in this paper on the KITTI dataset are shown in Figure 16. The KITTI dataset contains a total of 194 pairs of RGB images and 194 corresponding optical flow maps. We reviewed the converted IR images one-by-one and found that they visually approximated the real IR images.

On the basis of visual comparison, this paper also further compares the image generation results of the different networks using three evaluation metrics, namely peak signal-to-noise ratio (PSNR), structural similarity (SSIM), and root mean square (RMSE), with the real IR images used as the benchmark. Since the optical flow dataset KITTI is not an RGB-IR bimodal image dataset, in which the RGB images do not have corresponding real IR images, the evaluation experiments are conducted only on the training and test sets based on the M^3^FD division. Among the three metrics (Table 1), larger values of PSNR and SSIM indicate that the quality and similarity of the generated IR images are closer to the real IR images, while smaller values of RMSE indicate that the end-to-end pixel error between the generated IR images and the real IR images is minimized. The results reflected from the metrics data are largely consistent with the visual perception, with FDIT and AlignGAN2 based on unsupervised training performing poorly in all three metrics. On the other hand, Pix2Pix and ours, based on supervised training, maintain a high level on the training set while performing significantly better than the results of the unsupervised training on the test set.

### 4.2. Experimental Results of Optical Flow Computation

The training of RAFT and FastFlowNet is based on the RGB optical flow dataset KITTI (RGB KITTI) and IR KITTI generated by converting KITTI using the image transformation network. To verify the positive contribution of IR KITTI to the parameter fitting of the optical flow computation network in the IR scene, experiments were conducted using RGB KITTI. RGB KITTI + IR KITTI were trained on RAFT (or FastFlowNet) to obtain two optical flow computation networks labeled as RGB Network and IR Network to perform optical flow computations on real IR images and compare the differences between the computation results of the two networks.

The conventional practice for verifying the computational accuracy of a model is to conduct validation experiments with a dataset containing ground truth. And according to what has been mentioned above, none of the current optical flow dataset studies have been conducted by capturing IR image optical flows. Because of this realistic background, we propose a novel evaluation method. Briefly, we utilized the alignment of the RGB and IR images in the M^3^FD dataset in verifying the accuracy of the IR image optical flow computation. We evaluate the computational results by comparing the approximation of the optical flow computed for the IR image with the results on the RGB image. The optical flow of the RGB image, which is used as a benchmark for comparison, is obtained via supervised training on several public optical flow datasets. It is fine-tuned and verified on the real optical flow dataset, which has a strong confidence level and can be used as a strong evaluation index.

**Figure 17:**
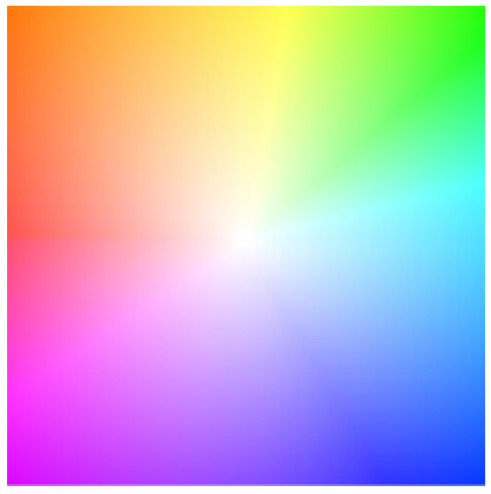
Benchmark of optical flow visualization. Different colors indicate different directions, and the intensity of each color represents the magnitude of the optical flow.

In addition, we use a reference color wheel (Figure 17) to visualize the optical flow results. The direction of the optical flow is expressed in different colors, while the size is equal to the pixel brightness size (pixel value) of the corresponding point in the visualized image.

**Figure 18:**
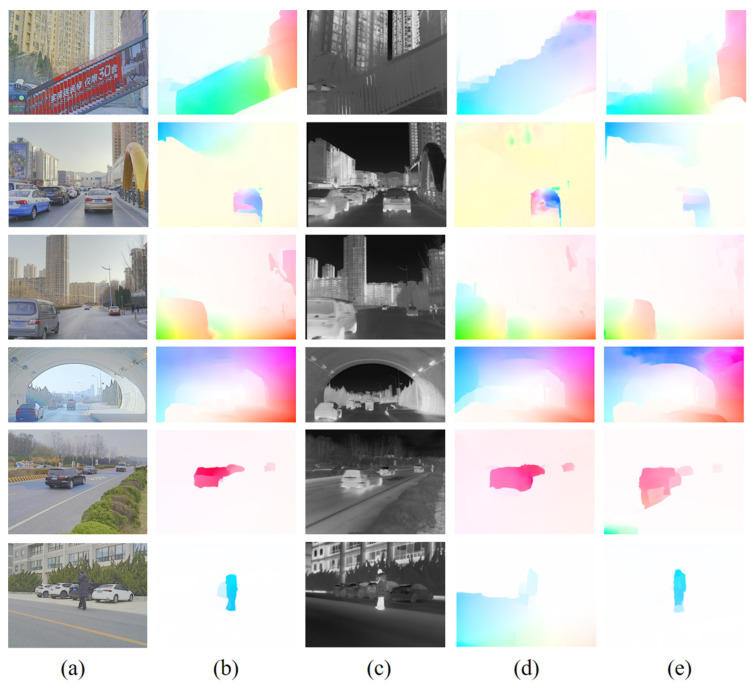
Computational results of optical flow computation networks (RAFT) trained on aligned RGB-IR with different training sets. (**a**) RGB image; (**b**) RGB Network calculates RGB image optical flow result; (**c**) IR image; (**d**) RGB Network calculates IR image optical flow result; (**e**) IR Network calculates IR image optical flow result.

**Figure 19:**
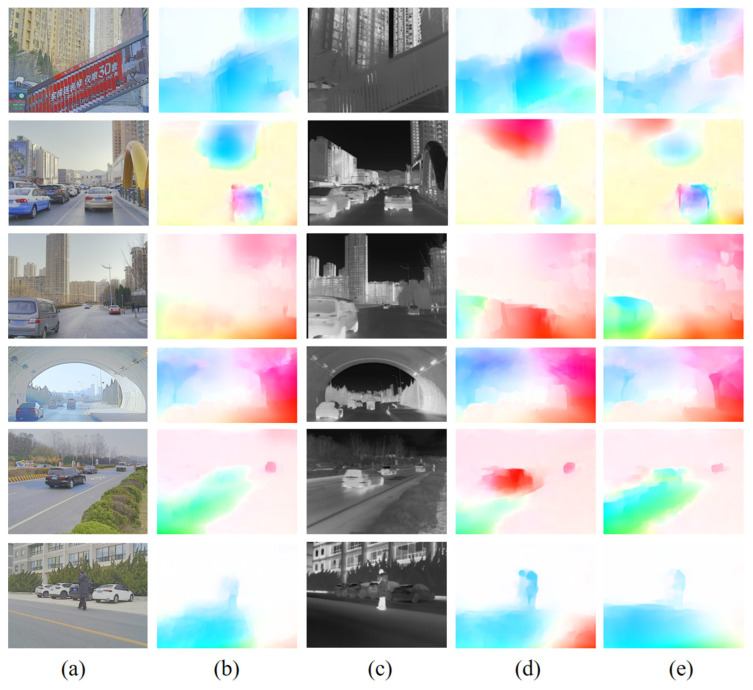
Computational results of optical flow computation networks (FastFlowNet) trained on aligned RGB-IR using different training sets. (**a**) RGB image; (**b**) RGB Network calculates RGB image optical flow result; (**c**) IR image; (**d**) RGB Network calculates IR image optical flow result; (**e**) IR Network calculates IR image optical flow result.

The results of the IR image optical flow computation experiments are shown in Figure 18 and Figure 19. Based on RGB and IR images (M^3^FD) alignment, the images shown in (b) in Figure 11 and Figure 12 are determined as the benchmarks for comparison of the IR image optical flow computation results, respectively. The images shown in (d) are the results of optical flow computation of the IR images by a network trained only using RGB images, and the images shown in (e) are the results of optical flow computations of IR images by a network further trained with generated IR images. The images in (d) and (e) are compared with benchmark (b), respectively. The visualized results of the optical flow from RAFT and FastFlowNet shown in (d) are not as accurate as (e) in terms of optical flow direction. The results shown in (e) are also more accurate in terms of optical flow size, and the intensity and distribution of the colors in the visualized image are closer to that of the benchmark (b). Applying the optical flow computation network trained using only RGB images directly to the optical flow computation of IR images does not maintain consistency and accuracy with the optical flow computation of RGB images. Additionally, the parameters of the optical flow computation network are more obviously overfitted to the RGB domain. Adding the IR network trained using IR KITTI overfitted the parameters of the network to the IR domain so that the results of the optical flow computation network trained for IR images were more consistent with the benchmark in terms of visualization.

Furthermore, following the judging criteria that have been used in the field of optical flow algorithms, we calculated the EPE (endpoint error) of the optical flow computation results (Table 2) of the network for the IR images before and after training using the generated IR images with respect to the benchmark. The corresponding visualization results shown in (d) and (e) correspond to the EPE of (b), respectively. The EPE results of RAFT are 64.8766 (without IR KITTI) and 49.3069 (with IR KITTI), and the EPE results of FastFlowNet are 8.6948 (without IR KITTI) and 7.5395 (with IR KITTI). Between RAFT and FastFlowNet, the difference in the results across the different networks is clearly due to the fact that the different networks have different complexities and different processing capabilities for the images. However, the more obvious and important argument is that in the same network, the IR images generated by the RGB-IR cross-modal network substantially improve the accuracy of the network’s computation of the optical flow of the IR images, achieving smaller errors in the comparison with the benchmark.

## 5. Discussion

Due to the great achievements of deep learning theory in the field of optical flow computation for RGB images in 2015, it is natural to wonder whether it can be applied to images of other modalities, such as IR images. According to the empirical formula, due to the naturally existing feature differences between RGB images and IR images, we will inevitably need to fine-tune the model for IR scenes when migrating to compute IR image optical flow.

Considering the production cost, in order to solve the problem of the IR optical flow dataset required for fine-tuning, this paper proposes the use of an RGB-IR cross-modal transformation network to augment the existing RGB optical flow dataset for IR scenes. We use the broadened IR data to perform parameter fine-tuning experiments on several optical flow computational networks, achieving more obvious results, with the fine-tuned networks achieving smaller errors in computing IR image optical flows. It is worth mentioning that there is still an essential gap between the simulation-generated IR images and real IR images. The production of real IR optical flow datasets can provide better training and testing benchmarks in the field of deep learning IR optical flow computation, which is a valuable contribution to scientific research.

## 6. Conclusions

In this paper, we propose the use of an RGB-IR cross-modal image transformation network to augment the existing RGB optical flow dataset with IR scenes. We then use the generated IR images to fine-tune the parameters of the optical flow computation network to improve the network’s optical flow computation accuracy for IR images. In addition, due to the lack of real infrared optical flow datasets, this paper utilizes the alignment of an RGB-IR bimodal dataset, M^3^FD, for the evaluation of the optical flow computation results of optical flow computation networks for IR images. We tested the optical flow computation results of the optical flow computation networks before and after adding the generated IR images for training on real IR images, and the EPE of the latter optical flow computation results was reduced by 24% (RAFT) and 13.29% (FastFlowNet).

Although the theory and experiments in this paper have yielded impressive results, some shortcomings still exist. The foremost problem that remains is the lack of real IR optical flow datasets. As far as the conditions and capabilities allow, the collection and production of IR optical flow datasets can better improve the training and validation experiments of optical flow computational networks in IR scenes. Furthermore, the RGB-IR cross-modal transformation network in this paper requires more research and refinement to generate more realistic IR images, which can also better contribute to the optical flow computation capability of the network.

## Figures and Tables

**Table 1:** Comparison of RGB-converted IR image results of different networks on the training set, test set, and KITTI dataset.

	Training Set			Test Set		
	PSNR	SSIM	RMSE	PSNR	SSIM	RMSE
Pix2Pix	38.6729	0.9772	0.0118	**21.6372**	**0.7048**	**0.1003**
FDIT	9.0525	0.3992	0.3658	9.5175	0.4217	0.3495
AlignGAN2	16.9659	0.6568	0.1457	14.5287	0.6030	0.1918
Ours	**40.0976**	**0.9797**	**0.0102**	20.9572	0.6915	0.1057

**Table 2:** Comparison of the optical flow computation results of the network for real IR images before and after training on hybrid generated IR images (based on the RGB-IR image alignment, assuming that the RGB-IR optical flow results are also aligned and using the optical flow computation results of the RGB image as a benchmark to calculate EPE, respectively).

	RAFT	FastFlowNet
RGB Network (without IR KITTI)	64.8766	8.6948
IR Network (with IR KITTI)	**49.3069**	**7.5395**
Percentage increase	24%	13.29%

## Data Availability

The RGB-IR cross-modal transition algorithm codes are available online at: https://github.com/ReggieBird/RGB2IR (accessed on 20 February 2024).
